# Fractal properties in sensorimotor variability unveil internal adaptations of the organism before symptomatic functional decline

**DOI:** 10.1038/s41598-019-52091-y

**Published:** 2019-10-31

**Authors:** Kjerstin Torre, Grégoire Vergotte, Éric Viel, Stéphane Perrey, Arnaud Dupeyron

**Affiliations:** 1 EuroMov, Univ. Montpellier, Montpellier, France; 2 CHU Carémeau, Nîmes, France

**Keywords:** Motor control, Time series

## Abstract

If health can be defined as adaptability, then measures of adaptability are crucial. Convergent findings across clinical areas established the notion that fractal properties in bio-behavioural variability characterize the healthy condition of the organism, and its adaptive capacities in general. However, ambiguities remain as to the significance of fractal properties: the literature mainly discriminated between healthy *vs*. pathological states, thereby loosing perspective on the progression in between, and overlooking the distinction between adaptability and effective adaptations of the organism. Here, we design an experimental tapping paradigm involving gradual feedback deprivation in groups of healthy subjects and one deafferented man as a pathological-limit case. We show that distinct types of fractal properties in sensorimotor behaviour characterize, on the one hand impaired functional ability, and on the other hand internal adaptations for maintaining performance despite the imposed constraints. Findings may prove promising for early detection of internal adaptations preceding symptomatic functional decline.

## Introduction

“What is health? The ability to adapt”^1^. Consistently, this has also been the basic assumption of a medical and translational research field taking a complex systems approach to medicine and motor behaviour for the past two decades^2–7^. However, conceptualizing health as the ability to adapt comes along with an acute need for defining *ad hoc* measurements of such adaptability^2,8^. Given the difficulty to appraise the intricate multiscale organization of complex systems, regardless of their nature, one approach has been to recognize that the manner in which some bio-behavioural variables fluctuate over time contains significant information about the condition of the organism itself. Based on this assumption, mounting evidence showed that unconstrained young and healthy organisms generate variables with highly complex –typically fractal– patterns of fluctuations^5,9–12^. In contrast, complexity is lost with pathologies or aging, conditions commonly associated with a loss of the organism’s adaptive capacities^5,6,13–15^. There are numerous examples, including heart failure^5^, neurodegenerative diseases like Parkinsons’s or Huntington’s^16^, attention deficit disorders^17^, depression^18^, aging and sensory deficit^19^. The convergence of results across such a wide range of clinical situations led researchers to investigate the diagnostic and prognostic power of time series complexity^20–22^. It also contributed to ground the idea that fractal complexity in the system observables is a hallmark of its adaptability^5,6,11,12,14^. While this idea is inspiring and conceptually plausible in various respects, accumulation of empirical findings is not evidence, and some significant issues remain.

Specifically, two interrelated conundrums arise from the literature. Firstly, if time series complexity is indeed a hallmark of organism’s adaptability as a cross-cutting factor, then this relationship is necessarily transposable to various circumstances outside of pathology or aging: one could then assume that experimentally-induced constraints mimicking pathology- or age-associated impairments would alter the complexity of functional variables in a similar way. The relative compartmentalization of the literature certainly contributed to leave this point out: on the one hand, clinical approaches mostly addressed the alteration of complexity due to a pathological state or aging via cross-sectional studies, or in relation to the level of impairment (*e*.*g*.^16,18,23,24^). Outside clinical conditions, only few studies did in fact investigate how fractal properties reflect the organism’s internal adaptations under experimental constraints while respecting task equivalence^25–27^. Secondly, at a specified functional level, adaptability embraces multiple complementary dimensions, in particular changeableness (the ability to exploit a wide range of the functional repertoire) and robustness (the capacity to maintain functional indifference in the face of changing conditions). In fact, adaptability strongly interferes with the processes of organism’s effective adaptations, whether evidenced at the functionally meaningful observation level or at any other level of the system. Furthermore, effective adaptations occurring at any level of the system may also affect further adaptability^28,29^. On account of these issues, one cannot ascertain whether an alteration of complexity in the organism’s variables reflects mechanisms specifically related to pathology, or the loss of adaptability associated with impaired conditions, or effective adaptations of the organism as a whole to maintain its functional level as far as possible when facing constraints.

An inclusive approach to complexity, adaptability, and adaptation in- and outside clinical conditions seems timely in order to define *ad hoc* measures of adaptability as a test to discriminate along a spectrum between healthy and pathological. One may consider that adaptive processes intervene in the organism up to a certain extent, after which any further adaptability is lost and functional impairment will eventually occur (*e*.*g*.^6^). This leads to rethinking fractal complexity as a slider in a continuum between healthy states and impaired pathophysiological states (*e*.*g*.^6,30^, see also^31^). Here, we propose an experimental finger-tapping paradigm allowing to preserve task performance under conditions of gradual sensory feedback (FB) deprivation in 69 healthy subjects (Controls, and deprived of 1, 2 or 3 FB). The suppressed sensory feedbacks were visual and/or auditory and/or somesthetic, using a sensory nerve block. One deafferented subject (I.W.) was involved as the pathological-limit case: I.W. presented with sensory neuropathy associated with loss of proprioceptive functions and sense of touch, and was additionally deprived of visual and auditory feedbacks. We analysed the mono- and multifractal properties of the series of inter-tap intervals produced. We expected that this experimental paradigm would contribute to unravel how fractal properties in bio-behavioural variability characterize adaptability, adaptation, and healthy or pathological.

## Results

For ease of understanding of the results, please note that each participant performed the task twice under the same condition of feedback deprivation, since it has been previously recommended to average the fractal exponents obtained from repetitions on a same task to improve the characterization of fractal properties (*e*.*g*.^32,33^).

### Tapping performance

For the two performance variables, *i*.*e*. Absolute Error (*AE* in ms) and Coefficient of Variation (*CV* in %) of the series of inter-tap intervals (ITI) produced, the repeated measures ANOVA 8(Sense) × 2(Trial) showed no significant Trial effect (*AE*: F(1, 61) = 0.042, *p* = 0.838; *CV*: F(1, 61) = 0.841, *p* = 0.363). Consequently, for these two trials, the values obtained per participant were averaged for each variable.

The nested ANOVA Sense(No.FB) showed neither an effect of the nested factor Sense (*AE*: F(4, 61) = 0.413, *p* = 0.798; *CV*: F(4, 61) = 1.606, *p* = 0.184), nor an effect of the nesting factor No.FB (*AE*: F(3, 61) = 2.492, *p* = 0.069; *CV*: F(3, 61) = 1.598, *p* = 0.199).

**Figure 1:**
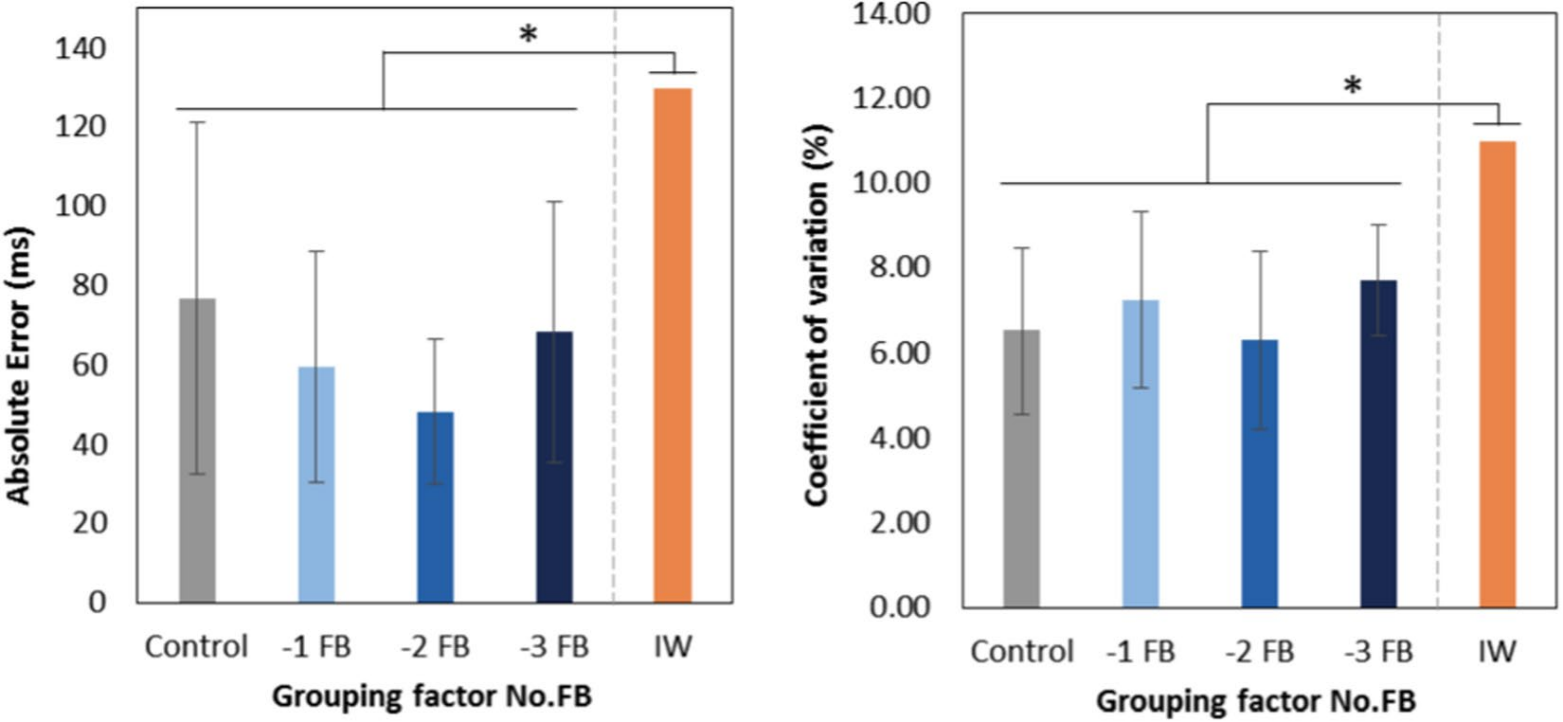
Performance variables for the four No.FB groups and I.W. Left: Absolute Error of ITI series with respect to target tempo. Right: *CV* of ITI series. The pattern of results is qualitatively the same for the two variables, with significantly altered tapping performance for I.W. compared to each of the four No.FB groups. Error bars represent standard deviation. *p < 0.05.

Finally, comparison with tapping performance by I.W. underlined significant differences between I.W. and each of the Control, −1 FB, −2 FB, and −3 FB groups for the two variables: the tapping performance for I.W. was globally altered, with larger *AE* (I.W. *vs*. Control: t(9) = 3.789, *p* = 0.004; I.W. *vs*. −1 FB: t(25) = 12.301, *p* = 0.000; I.W. *vs*. −2 FB: t(22) = 21.378, *p* = 0.000; I.W. *vs*. −3 FB: t(9) = 5.930, *p* = 0.000) and more variable tapping intervals (CV: I.W. *vs*. Control: t(9) = 7.204, *p* = 0.000; I.W. *vs*. −1 FB: t(25) = 9.858, *p* = 0.000; I.W. *vs*. −2 FB: t(22) = 10.767, *p* = 0.000; I.W. *vs*. −3 FB: t(9) = 7.871, *p* = 0.000). Figure 1 summarizes tapping performance results.

### Monofractal properties of tapping series (*α*)

**Figure 2:**
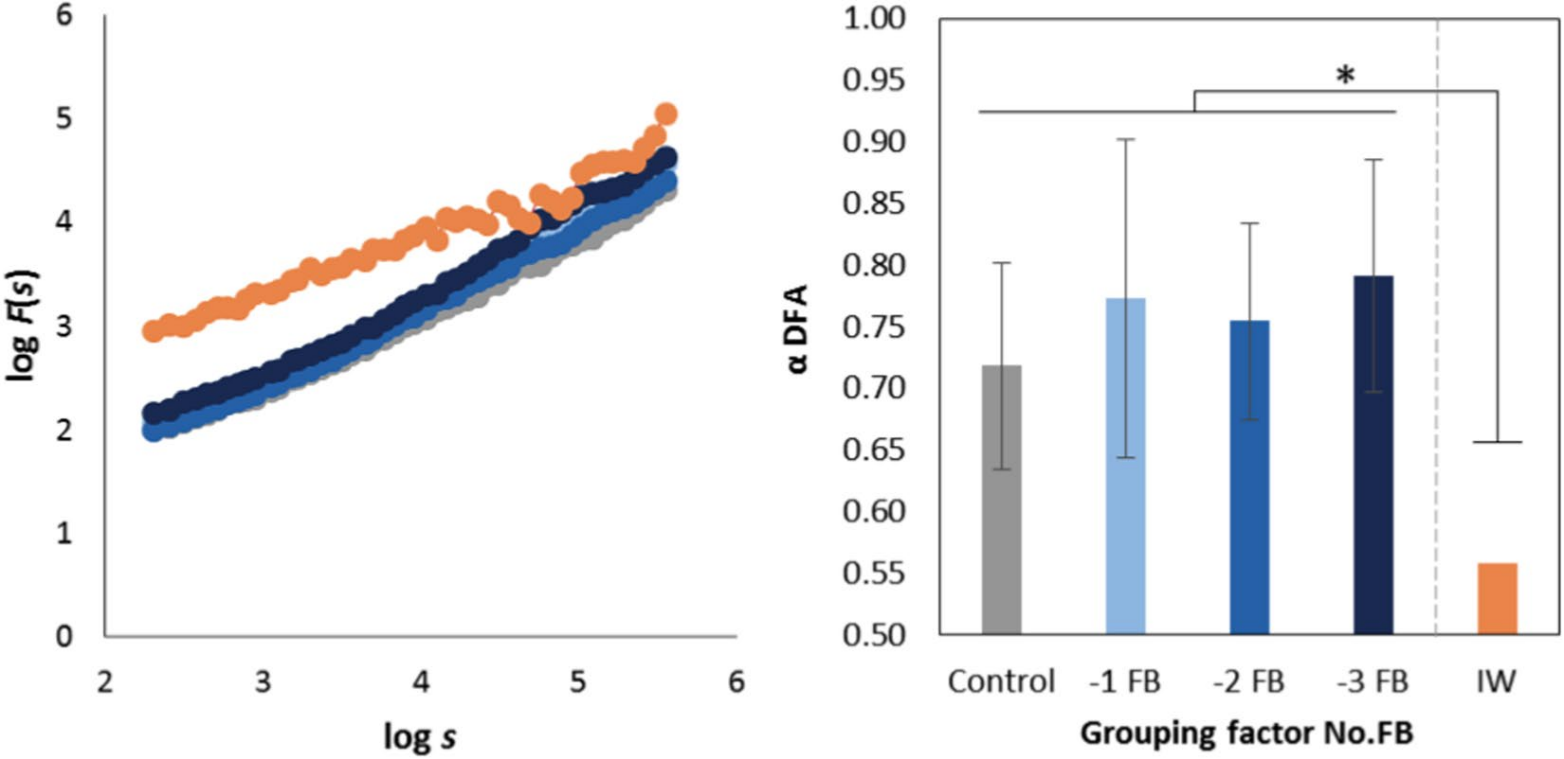
Results on monofractal properties. Left: Average diffusion plots in log-log coordinates obtained from DFA for the four No.FB groups and I.W. The monofractal exponents *α* correspond to the slopes of the regression lines fitting the plots. Right: Average fractal exponents; *α* is significantly lower for I.W. than for each of the four No.FB groups, and tends towards 0.5 (*i*.*e*., white noise). Error bars represent standard deviation. *p < 0.05.

All participants and series considered, the evenly spaced Detrended Fluctuation Analysis (DFA)^34–36^ yielded monofractal exponents *α* ranging from 0.42 to 1.12. For 135 out of the 140 ITI series (96%), DFA yielded *α* < 1. The ITI series produced were thus consistently characterized as fGn. Figure 2 displays the average diffusion plots for the Control, −1 FB, −2 FB, and −3 FB groups, and for I.W. Statistical analyses applied to α revealed a similar pattern of results as obtained for the tapping performance: the repeated measures ANOVA 8(Sense) × 2(Trial) showed no significant Trial effect (F(1, 61) = 1.352, *p* = 0.249), and similarly the nested ANOVA Sense(No.FB) did not show an effect of Sense (F(4, 61) = 0.045, *p* = 0.996) nor of No.FB (F(3, 61) = 0.950, *p* = 0.422). However, comparison between I.W. and the Control, −1 FB, −2 FB, and −3 FB groups revealed that *α* was significantly lower for I.W. than for each of the four groups (I.W. *vs*. Control: t(9) = 5.999, *p* = 0.000; I.W. *vs*. −1 FB: t(25) = 8.478, *p* = 0.000; I.W. *vs*. −2 FB: t(22) = 11.802, *p* = 0.000; I.W. *vs*. −3 FB: t(9) = 7.829, *p* = 0.000). Figure 2 summarizes the results obtained for the monofractal properties of ITI series.

### Multifractal properties of tapping series (*MF-Width*)

**Figure 3:**
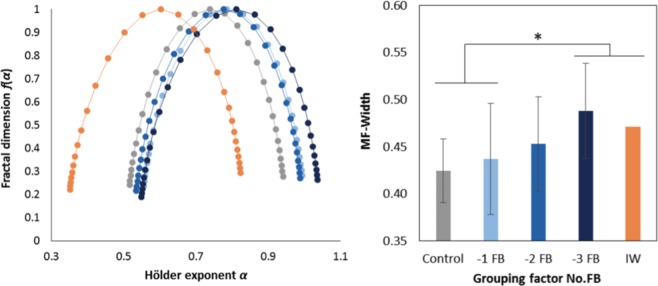
Results on multifractal properties. Left: Average singularity spectra obtained from MF-DFA for the four No.FB groups and I.W. The width of a spectrum corresponds to the range between the highest and lowest singularity exponents (*α*). Right: Average multifractal widths; *MF-Width* for the −3 FB group and I.W. do not significantly differ, but both are larger than for the Control and −1 FB groups. Error bars represent standard deviation. *p < 0.05.

All participants and ITI series considered, the Multi-Fractal Detrended Fluctuation Analysis (MF-DFA)^37,38^ yielded singularity spectra with widths (*MF-Width*) ranging from 0.34 to 0.58. Figure 3 displays the average multifractal spectra for the Control, −1 FB, −2 FB, and −3 FB groups, and for I.W. The repeated measures ANOVA 8(Sense) × 2(Trial) on *MF-Width* showed no significant Trial effect (F(1, 61) = 0.841, *p* = 0.363). The nested ANOVA Sense(No.FB) showed no effect of the nested factor Sense (F(4, 61) = 0.399, *p* = 0.809), but did show an effect of the nesting factor No.FB (F(3, 61) = 2.955, *p* = 0.039): post-hoc analysis showed that *MF-Width* was significantly higher for the −3 FB group than for the Control (*p* = 0.009) and −1 FB groups (*p* = 0.01). Comparisons with I.W. showed that *MF-Width* was also significantly higher for I.W. than for the Control (t(9) = 4.401, *p* = 0.002) and −1 FB groups (t(25) = 2.969, *p* = 0.006), but not different from the −3 FB group (t(9) = 1.032, *p* = 0.329). Figure 3 summarizes the results obtained for the multifractal properties of ITI series.

Finally, all participants considered, Spearman’s rank correlation revealed a significant monotonic and positive relationship between *MF-Width* and the number of feedback suppressed (*ρ* = 0.37, *p* = 0.002).

## Discussion

We addressed the challenge of clarifying the significance of fractal complexity in terms of adaptation, adaptability, pathology and health. Our main results were (i) no significant effect of feedback deprivation on tapping performance in neurologically intact groups, but decreased performance in I.W.; (ii) an increased degree of multifractality of tapping series when increasing the number of feedbacks suppressed; (iii) a decreased monofractal exponent in I.W. compared to all healthy groups.

### Eliciting internal adaptations independently of functional impairment and pathology

Results showed no significant alteration of tapping performance regardless of the number and nature of the feedbacks suppressed in healthy subjects. These results are thus consistent with the idea that no sensory feedback is essential in tapping, since multiple adaptive configurations can produce comparable tapping performance depending on circumstances. Indeed, as regards to feedback deprivation in self-paced tapping, there is no firm consensus on the relative contribution and integration of sources of sensory information. For a long time, self-paced tapping was mainly considered open-loop, as formalized by the well-established Wing & Kristofferson model^39^. However, research has since shown that several feedback manipulations could likely elicit compensatory patterns in timing behaviour, thus indicating some form of sensory integration in tapping performance^40–43^. In fact, as evidenced in the literature it seems that tapping performance could be regulated by several configurations of interrelated subsystems, or strategies (*e*.*g*.^44,45^): while the “minimal” open-loop configuration may enable to carry out consistent tapping performance under circumstances, proprioceptive, auditory, and visual feedbacks provide partially redundant information integrated depending on availability/salience and the various constraints imposed to the bio-behavioural system.

As regards to our pathological limit case with chronic deafferentation, patients have likely developed compensatory strategies, including increased reliance on visual and auditory feedbacks^44,46,47^, allowing to produce reasonably consistent self-paced tapping, however without attaining the same performance as control subjects^41,44^. Our results from I.W. – deprived of auditory and visual feedbacks in addition to his intrinsic loss of somesthetic feedback – were congruent: tapping performance was not dramatically impaired in I.W. however it was significantly deteriorated compared to the healthy groups, with increased variability and lengthened inter-tap intervals (Fig. 1). According to the literature, the age factor (I.W. aged 61 years at the time of the study) might have contributed to the global slowing of the tapping tempo but is unlikely the cause of the increased variability^41,48^.

In light of the above elements, our experimental setting appears heuristic. Indeed, experimental feedback deprivation in healthy groups allowed for unaltered performance without changing the nature of the task, thanks to internal adaptations. Moreover, feedback deprivation could be experimental *vs*. pathological in nature, and both allowed for consistent and exploitable performance on the same task, while revealing functional impairment in the pathological subject. However, some limitations to the study should be taken into account. Firstly, none of the healthy groups showed any significant decrement in performance. Hence, it was impossible to identify some contrasts, in particular comparing the effects of pathological *vs*. non-pathological functional impairments on fractal properties. Secondly, it is worth recalling that a compromise between ideal and feasible methodological rigor, as regards notably times series length, is unavoidable when assessing behavioral data collected from patients or under constraining conditions. Aware of this, we took care to perform analyses on satisfying sample sizes, worked on repeated performances by each participant on the same task, and integrated recent methodological developments of mono- and multifractal analyses proposed in the literature, notably evenly spacing and the focus-based approach^35,38^. Finally, although the −3 FB group and I.W. were intended to be formally comparable in terms of the number of feedbacks suppressed, the nature and range of internal adaptations and neuroplastic changes associated with localized transient *vs*. complete chronic conditions were obviously not comparable^49,50^. While such limitation applies to the present study, it does also impede experimental efforts to clarify the meaning of fractal properties in terms of pathological *vs*. non-pathological constraints.

### Multifractality – a marker of internal adaptations of the organism

Suppression of one or other of the visual, auditory, or somesthetic feedbacks had no differential effect on the multifractal properties of the tapping series. In contrast, when increasing the number of feedbacks suppressed in the healthy groups (regardless of the sensory modalities) the width of multifractal spectra increased, while tapping performance remained statistically unchanged. Although differences in *MF-Width* did not reach significance for all No.FB comparisons, correlation analysis showed that the degree of multifractality was linearly related to the number of feedbacks suppressed. *MF-Width* did not significantly differ between I.W. and the healthy −3 FB group, showing that pathology *per se* – *i*.*e*. once controlled for the number of feedbacks suppressed – had no specific effect on the degree of multifractality.

Unlike monofractal properties, multifractal properties in significant functional variables quantify interaction-dominant (as opposed to component-dominant) dynamics in complex systems’ behaviours; they encapsulate the nonlinear cross-scale interactions among the multiple subsystems in the organism, and are well accounted for by multiplicative cascading models^51,52^. So, multifractals likely characterize the coordination among subsystems, perceptual or others, and putative shifts in how the system regulates to adapt to changing constraints (*e*.*g*.^25,53^). In this line, multifractal fluctuations in movement variables were also shown to predict the integration and use of perceptual information to adapt motor behaviour to external perturbations^25^. More specifically, recent studies bridging behavioural and brain observation levels showed that the degree of multifractality in tapping relates to (re)organizations in functional brain connectivity underlying the tapping performance: an increase in the width of multifractal spectra of ITI series was shown to correlate with increased number of different brain networks involved in the task^54^. Shifts in brain connectivity were indeed evidenced as an adaptive response to feedback deprivation in the cases of chronic/pathological conditions as well as transient experimental manipulations, with notably an increased connectivity and diversification of neural circuits across sensory and non-sensory areas^49,50,55,56^. Based on these elements, our findings of increased multifractality appear fully consistent with an increased repertoire of interactivity within the organism^51,57^ as an adaptation to experimentally- or pathology-induced feedback deprivation.

Showing that alterations of fractal properties enable to identify subtle changes in the systems’ organization or condition when the functional ability does not yet exhibit it, is not a new finding *per se* (*e*.*g*.^16^). However, previous evidence often stemmed from cross-sectional studies, comparing pathological or elderly subjects to healthy/young controls. As a matter of fact, it was suggested that alterations of (multi)fractal properties in the organisms observables reflect their impaired/pathological condition, and by extension a loss of their adaptive capacities. If we had focused on I.W. and the control group only, it would have been tempting to reach the same conclusion. However, our work takes a step further since it reveals a step-by-step evolution of multifractality irrespective of functional impairment and pathology, as a function of the gradual constraints imposed to healthy participants – up to I.W. as our pathological-limit case. Accordingly, we argue that multifractality reflects internal adaptations of the organism to maintain when facing constraints a given functional level, whether in pathological or healthy subjects. By uncovering necessary internal adaptations of the organism ahead of any apparent functional impairment, multifractal properties could thus be particularly promising in the perspective of developing early diagnostic/prognostic measures of pathological states before their clinical onset.

### Monofractal properties – a marker of impaired functional ability and/or pathology

Neither the factor sensory modality nor the factor number of feedbacks suppressed had an effect on the monofractal properties of tapping series in healthy groups. Remarkably however, I.W., deprived of visual and auditory feedbacks in addition to his pathological loss of somesthetic feedback, showed an *α* exponent close to 0.5 (*i*.*e*., increased randomness) and significantly lower than for all the healthy groups, including the group −3 FB (AVS). This pattern suggests that the drop of the monofractal exponent is not due to the number of feedbacks suppressed, but to the intrinsic pathological condition in I.W.

These results may be considered together with a previous study by Manor *et al*.^19^ where four groups of elderly subjects presenting with (i) no sensory impairments, (ii) visual impairments only, (iii) somatosensory impairments only, and (iv) combined impairments, performed a quiet standing task. The complexity of postural sway was found to decrease gradually from controls to the group with combined feedback impairments. At a first glance our present results could seem incongruent, since one might have anticipated a gradual decrease of complexity with an increasing number of feedbacks suppressed from controls to the −3 FB group. However, an important point is that in Manor *et al*.^19^, the gradual loss of complexity across groups paralleled with a gradual deterioration of classical sway parameters (sway area and velocity), and that the alteration of feedbacks was systematically due to the impaired condition of the organism. That is, the level of constraints on the system, altered functional performance, and pathology remained putative confounded factors in time series complexity. Our results allow to go one step beyond in this respect: the loss of complexity in I.W. but in none of the experimental −1, −2, and −3 feedback deprivation groups suggests that adaptations facing increasing constraints on the system is not a determinant of the monofractal properties in output variables.

Nonetheless, the present results on monofractal properties are not fully conclusive. Indeed, since none of the healthy groups showed any decrease in performance with feedback deprivation while I.W. presented with both a pathology and impaired performance, we were not able to ascertain whether the loss of fractal complexity was related to impaired functional ability regardless of its origin, or was specifically associated with pathological impairments. At this stage, one could thus consider than an alteration of monofractal properties does not reflect internal adaptations but indeed the limit of such adaptations, *i*.*e*. loss of adaptability. While this idea matches with previous literature^5,6,14^, it should however be handled with caution in regards to two aspects: first, it does not constitute an evidence for the thesis of loss of complexity with aging and disease, since it remains unclear whether the relationship also holds outside any clinical context (*e*.*g*.^13,58^). Second, underlining complexity as a hallmark of adaptability poses a broader issue: adaptability is conceptually vague, as it encompasses multiple dimensions (for example indifference to changing constraints, the repertoire of possible behaviours, anticipation of changing constraints, etc.)^59^, and unfolds across several contexts and observation levels (*e*.*g*., mental flexibility, brain plasticity, etc.). This makes the complexity-adaptability relationship hardly testable/falsifiable other than by using default reasoning. In the current stage of knowledge, the commonly assumed association of complexity, in particular fractal properties, and adaptability appears to be a shortcut that might prove an epistemological obstacle under circumstances.

## Conclusion

The present experimental approach of gradual feedback deprivation in between the two – healthy unconstrained and pathological – extremes, promotes the idea of a continuum integrating internal adaptations into the intricate picture of adaptability, functional impairment, and pathology. Our results on monofractal properties converge with the idea that loss of time series complexity reflects a loss of adaptive capacities of the organism. Future research should clarify whether it is pathology-specific by considering different aspects of adaptability outside clinical contexts. In contrast, we argue that multifractal properties reflect internal adaptations of the organism under constraints, being sensitive to changes in the systems interactions even in the absence of functional alteration. As such multifractal properties could be particularly promising for developing early diagnostic/prognostic measures of pathology ahead of objective functional decline.

## Materials and Methods

### Subjects

Seventy subjects, 69 young and healthy individuals (38 males, age 24.3 ± 5.0 years, 66 right-handed) and 1 subject presenting with chronic peripheral deafferentation, I.W. (male, age 61 years, left-handed), were included in the study. Healthy subjects were included if aged between 18 and 45 years and meeting none of the following exclusion criteria: (i) pregnant, parturient, or nursing women; (ii) neurological pathology affecting the central or peripheral nervous system; (iii) recent or current trauma of the upper limb; (iv) severe and uncorrected visual or auditory impairment; (v) intensive musical training. Subjects signed an informed consent after a medical consultation, and the experiment was performed in accordance with all relevant guidelines and regulations. The procedures were approved by the Ethics Committee Sud Méditerrannée III (CPP no. 2015.09.01).

I.W. presents with peripheral deafferentation with selective loss of large myelinated sensory fibres yielding to a deficit of somesthetic feedback below the neck, specifically the sense of touch, pressure, and proprioception of joint positions and movement of the upper limbs. However, perception of pain and temperature remain unaffected. Motor nerves are also unaffected, as shown by normal motor nerve conduction velocities and electromyography results. Since the onset of this pathology at the age of 19 years, I.W. achieved good motor control relying particularly on vision (for details on disease characteristics see^60–62^). In regards to our purpose, the behaviour of I.W. offered a rare opportunity to compare the effects of deafferentation due to an extreme pathological condition *versus* experimentally-induced deafferentation in healthy subjects, on the fractal properties of outcome variables.

### Apparatus

Participants were sitting on an adjustable chair, with their dominant side forearm and hand resting on a customized plinth on a table in front of them. Previous tapping studies in I.W. reported a potential loss of contact with the tapping surface (the hand wandering in the air) for lengthy tapping sequences, due to absence of proprioceptive feedback (*e*.*g*.^40^). Therefore the hand was maintained on the surface during trials for I.W. A PC-driven auditory metronome (*Matlab* 2008a) delivered a sequence of 20 tones at a specified frequency. During the tapping task, movements of the index finger were collected using a small single-axis accelerometer stuck on the nail. Acceleration data were collected using a Labjack U12 device and stored via its software (LJStream v1.07). The sampling rate was 300 Hz. Depending on the experimental condition they were assigned to, participants wore a sleeping mask to prevent visual feedback, and/or noise suppression ear defenders to prevent auditory feedback, and/or they were administered a peripheral nerve block at wrist level to prevent somesthetic feedback.

### Experimental task and procedures

Subjects performed a finger tapping task following a classic synchronization-continuation paradigm^39^: during the initial phase participants had to tap in synch with 20 pacing signals delivered by the auditory metronome. The tempo imposed by the metronome was 1.5 Hz, known as a comfortable tapping frequency^63^. Once the metronome stopped, participants had to continue tapping as regularly as possible at the same tempo for 6 minutes 40 seconds. The trial duration was set to yield series of at least 512 ITI for subsequent fractal analysis^32^, while avoiding as much as possible putative effects of fatigue or weariness.

After inclusion, healthy participants were randomized into 8 experimental groups. The groups differed by the deprived sensory feedback(s), according to a two-factor nested design with the factor “Sensorial Modalities” (Sense) nested within the “Number of Feedbacks” (No.FB): each level of the factor Sense was found in combination with only one level of the factor No.FB. Sensory modalities manipulated were auditory (A), visual (V), and somesthetic (S) feedbacks. Previous studies showed that tapping behaviour was sensitive to experimental feedback manipulations, yet suppression of different sensorial feedbacks does not jeopardize consistent tapping performance (*e*.*g*.^47,64^). We suppressed sensory feedbacks in a single setting or in all possible combinations, thus yielding 8 levels for the nested factor Sense (no feedback, A, V, S, AV, SA, SV, and AVS suppressed), and 4 levels for the nesting factor No.FB (−0 FB/control, −1 FB, −2 FB, and −3 FB). Table 1 summarizes the experimental design. Such design thus allowed us to focus on the effect of the number of deprived feedbacks (with n = 10, 26, 23, and 10 for the control −1 FB, −2 FB, and −3 FB groups, respectively) while checking for putative differences due to the sensorial modalities concerned. Each participant performed two trials in the assigned condition, with a 2-minute rest in between.

**Table 1:** Summary of the experimental design.

	Number of feedbacks suppressed								
	Control	−1			−2			−3	−3
A		×			×	×		×	×
V			×		×		×	×	×
S				×		×	×	×	×
	**Healthy groups**								**IW**

A: auditory, V: visual, S: somesthetic.

I.W. performed two trials following the same procedure, being experimentally deprived of both auditory and visual feedbacks in addition to his intrinsic impairment of somesthetic feedback.

### Sensory nerve block

A locoregional anaesthesia was performed by injection of 2 ml of ropivacaïne 7.5 mg/ml at the wrist, at the contact of the ulnar, median, and radial nerves. At such low doses, the injection leads to a sensory block without affecting motor command. After injection, participants rested for 20 to 30 minutes to achieve the anaesthetic effect. In the case the effect was judged incomplete, an additional dose was administered at the site of the concerned nerve. Use of ropivacaïne at the doses used provides anaesthesia for 6 to 10 hours, and each participant performed the experimental task within 2 hours after injection.

### Data analysis

#### Pre-processing

Raw acceleration data were processed using *Matlab* 2016a. An algorithm for peak detection was applied to acceleration data to determine the tapping times, and series of ITI were computed as the difference between consecutive tapping times. Per trial, series of 512 ITI were retained for subsequent analyses.

#### Tapping performance

To assess performance precision we determined the absolute error (*AE* in ms) of ITI series in regards to the required target intervals (666 ms). To get a global assessment of the combination of all sources of variability commonly considered in tapping (*i*.*e*., motor and timekeeper variability) we calculated the coefficient of variation (*CV* in %) of ITI series.

#### Monofractal properties of tapping series

Linearly detrended ITI series were submitted to evenly spaced Detrended Fluctuation Analysis (DFA)^34^. To investigate the organization underlying human bio-behavioral variables, DFA is one of the most widespread and robust methods that characterizes the monofractal properties of time series. Fractal time series typically exhibit fluctuations with scale invariant structure (*i*.*e*., obeying a power law distribution

$$X(ct)={c}^{H}X(t),$$

where *X* is the signal, *c* is a constant, *H* is the fractal exponent) and temporal long-range correlations (meaning the autocorrelation function of the time series decays as a power-law without falling to zero). In short, DFA exploits the diffusion properties of time series, analysing the relationship between the characteristic size of fluctuations *F*(*s*) and the segment length *s* for which these fluctuations are measured. As a first step, the series *x*_*i*_ of length *N* is integrated by cumulative summation after subtracting the mean of the whole signal:

1 $$X(k)=\mathop{\sum }\limits_{i=1}^{k}[{x}_{i}-\frac{1}{N}\mathop{\sum }\limits_{i=1}^{N}{x}_{i}],\,\,k=1,\,\ldots ,N$$

The integrated series *X*(*k*) is then divided into *n* non-overlapping segments of length *s*, with *s* typically ranging from 10 to *N*/2. Within each segment, the series *X*(*k*) is linearly detrended by subtracting the theoretical values *X*(*k*)^*Th*^ given by the regression. Finally, for all possible segments lengths *s*, the characteristic size of fluctuations is computed as:

2 $$F(s)=\sqrt{\frac{1}{N-ks}\,\mathop{\sum }\limits_{k=1}^{N-ks}{(X(k)-{X}^{Th}(k))}^{2}}$$

For fractal series, a power-relationship characterized by the monofractal exponent *α* ∈ [0, 2] is expected:

3 $$F(s)\propto {s}^{\alpha }$$

**Figure 4:**
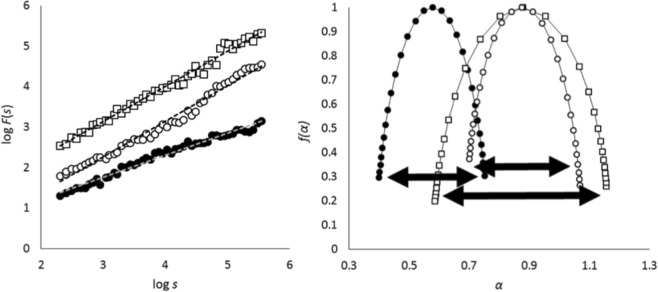
Illustration for mono- and multifractal methods. Left: DFA diffusion plots for three time series. The two white plots have nearly the same slopes (similar *α* exponents for the time series) while the black plot has a flatter slope (lower *α* exponent). Right: Singularity spectra for the same three time series. The shift to the left of the black spectrum corresponds to the relatively lower *α* exponent yielded by the DFA for this series compared to the two others. While for the two white spectra the central tendencies are nearly superimposed, the width of the square-dot spectrum is larger than for the circled one, showing that the two time series have different degrees of multifractality regardless of their monofractal exponent.

Exponent *α* thus corresponds to the slope of the plot *F*(*s*) against *s* in bi-logarithmic coordinates (see Fig. 4 for illustration). Recent developments of the methods addressed the points of the diffusion plot to be considered for the linear regression: evenly spacing (*i*.*e*. geometrically equidistant points of the plot instead of logarithmically spaced points) was shown to increase the reliability of *α* estimates^35,36^. We thus included this procedure in the present analysis.

By yielding a single fractal exponent that characterizes the time series, DFA assumes that the fractal properties are homogeneous over all time scales of the entire series. For 0.5 < *α* < 1 the series is a stationary fractional Gaussian noise (fGn) and contains persistent long-range correlations^65^. In particular, for *α* = 0.5 the series is white noise (random, totally unpredictable behaviour), for *α* = 1.5 the series is Brownian motion (regular, predictable behaviour), and for *α* = 1 the series is so-called 1/*f* noise. Complexity is considered maximal for 1/*f* noise, and decreases as *α* departs from 1, tending either towards 0.5 (white noise) or towards 1.5 (Brownian motion). Loss of complexity in both directions was found in the literature (*e*.*g*.^5,17^) though alterations towards white noise were far more commonly reported in the translational literature on human physiology and motor control.

#### Multifractal properties of tapping series

While monofractal analyses assume that the fractal properties are homogeneous over all time scales of the series, the temporal fluctuations of bio-behavioural variables are often heterogeneous^51^. Multifractal analyses are more fine-grained and allow to capture a combination of different intermittent fluctuation patterns, meaning putative variations of the fractal scaling properties over different time scales of the series. As such, multifractal analyses likely capture the organization and reorganizations among multiple subsystems that interact across various time scales to contribute to the global system’s performance (*e*.*g*. 25).

In particular, Multifractal Detrended Fluctuation Analysis (MF-DFA) was introduced by Kantelhardt *et al*.^37^, and received recent methodological developments. The first steps of the procedure are basically the same as for DFA. After integration of the original series *x*_*i*_ into *X*(*k*), segmentation of *X*(*k*) into *n* non-overlapping segments of length *s*, and linear detrending within each segment, the variance is determined for each segment:

4 $${F}^{2}(s,n)=\frac{1}{s}\mathop{\sum }\limits_{k=1}^{s}{\{X[(n-1)s+k]-{X}_{n}^{Th}(k)\}}^{2},\,\,\,n=1,\,\ldots ,{N}_{s}$$

The average size of fluctuations is then computed over all segments of equal length for *q*^th^ order moments, with *q* ranging from −15 to +15 in steps of 1 in the present case, to obtain the *q*^th^ order fluctuation function:

5 $${F}_{q}(s)={\{\frac{1}{{N}_{s}}\mathop{\sum }\limits_{n=1}^{{N}_{s}}{[{F}^{2}(s,n)]}^{q/2}\}}^{1/q}$$

Note that the particular case *q* = 2 corresponds to standard DFA computation.

If the series *x*_*i*_ has fractal scaling properties, *F*_*q*_(*s*) is scaled to *s* following a power law:

6 $${F}_{q}(s)\propto {s}^{h(q)}$$

The function *h*(*q*) is the generalized Hurst exponent, and for series that are fGn *h*(2) corresponds to the *α* exponent yielded by DFA. While for monofractal series *h*(*q*) is indent of *q*, in multifractal series *h*(*q*) describes different scaling behaviors for segments of the series with larger (for *q* > 0) and smaller (for *q* < 0) fluctuations.

From the above function, results can be converted into a standard multifractal formalism using simple transformations, to be finally summarized in the singularity spectrum (see^37^ for details on the mathematical transformations). The singularity spectrum relates the Hölder exponent *α*, representing the singularities of the scaling behaviours of the series, and the fractal dimension *f*(*α*) for each subset of the series characterized by the same Hölder exponent. One can relate the Hölder exponent *α* and *f*(*α*) to *h*(*q*) by:

7 $$\alpha =h(q)+qh^{\prime} (q)\,{\rm{and}}\,f(\alpha )=q[\alpha -h(q)]+1$$

The singularity spectrum thus captures the heterogeneity, or intermittent changes in scaling behaviours within the series^51^, and is expected to present a parabolic shape. The variable of interest here is the width *α*_*max*_ − *α*_*min*_ of the singularity spectrum (*MF-Width*), which represents the degree of multifractality of the series (Fig. 4).

It was reported that for experimental time series MF-DFA computation may sometimes yield “inversed” spectra instead of the expected parabolic shape^38,66^. To overcome this limitation, recent methodological developments by Mukli *et al*.^38^ have introduced the so-called focus based approach, using a theoretical focus of the scaling function *F*_*q*_(*s*) to guide the regression for *h*(*q*) (procedure relative to Eqs 5 and 6). We integrated this procedure in our present analysis since it was shown to allow for more robust performance and avoiding inversed spectra^38^.

### Statistical analyses

The statistical procedure was the same for all dependent variables (AE, CV, α, and *MF-Width*). Samples were first checked for normality and homogeneity of variances before applying parametric analyses. A 2-way repeated measures ANOVA Sense × Trial was then performed to check for a significant effect or the factor Trial. If there was none, we averaged the values obtained from trial 1 and trial 2 for each participant and applied a 2-way nested ANOVA Sense(No.FB). Likewise, values obtained from trial 1 and trial 2 with I.W. were averaged and compared as a test value to the four No.FB groups, using one sample *t*-tests. Finally, based on the results obtained from the nested ANOVA on *MF-Width*, we tested for a correlation between *MF-Width* and the number of feedbacks subjects were deprived of, using Spearman’s rank correlation. The significance threshold was set at *p* < 0.05.

## Data Availability

The datasets generated and analysed during the current study are available from the corresponding author on reasonable request.

## References

[1] Books Z (2009). What is health? The ability to adapt [Editorial]. The Lancet.

[2] Huber M (2011). How should we define health?. BMJ.

[3] Ahn AC, Tewari M, Poon CS, Phillips RS (2006). The limits of reductionism in medicine: could system biology offer an alternative?. PLoS Med..

[4] Goldberger AL (1996). Non-linear dynamics for clinicians: chaps theory, fractals, and complexity at the bedside. The Lancet.

[5] Goldberger AL (2002). Fractal dynamics in physiology: alterations with disease and aging. Proc. Natl. Acad. Sci. USA.

[6] Lipsitz LA (2002). Dynamics of stability: the physiologic basis of functional health and frailty. J. Gerontol. A Biol. Sci. Med. Sci..

[7] Sturmberg JP, Martin CM, Katerndahl DA (2014). Systems and complexity thinking in the general practice literature: an integrative, historical narrative review. Ann. Fam. Med..

[8] Cavanaugh JT, Kelty-Stephen DG, Stergiou N (2017). Multifractality, Interactivity, and the Adaptive Capacity of the Human Movement System: A Perspective for Advancing the Conceptual Basis of Neurologic Physical Therapy. J. Neurol. Phys. Ther..

[9] Van Orden, G. C., Kloos, H. & Wallot, S. Living in the pink: Intentionality, wellbeing, and complexity. Philosophy of complex systems. *Handbook of the philosophy of science*, ed. Hooker A (Elsevier, Amsterdam), pp. 639–684 (2010).

[10] Linkenkaer-Hansen K, Nikouline VV, Palva JM, Ilmoniemi RJ (2001). Long-range temporal correlations and scaling behavior in human brain oscillations. J. Neurosci..

[11] Sturmberg JP, Bennett JM, Picard M, Seely AJE (2015). The trajectory of life. Decreasing physiological network complexity through changing fractal patterns. Front. Physiol..

[12] Werner G (2010). Fractals in the nervous system: conceptual implications for theoretical neuroscience. Front. Physiol..

[13] Manor B, Lisitz LA (2013). Physiologic complexity and aging: Implications for physical function and rehabilitation. Prog. Neuropsychopharmacol. Biol. Psychiatry.

[14] Goldberger AL, Peng C-K, Lipsitz LA (2002). What is physiologic complexity and how does it change with aging and disease?. Neurobiol. Aging.

[15] Stergiou N, Decker LM (2011). Human movement variability, nonlinear dynamics, and pathology: is there a connection?. Hum. Mov. Sci..

[16] Hausdorff JM (1997). Altered fractal dynamics of gait: Reduced stride-interval correlations with aging and Huntington’s disease. Journal of Appl. Physiol..

[17] Gilden DL, Hancock H (2007). Response variability in attention-deficit disorders. Psychol. Sci..

[18] Leistedt SJJ (2011). Decreased neuroautonomic complexity in men during an acute major depressive episode: analysis of heart rate dynamics. Transl. Psychiatry.

[19] Manor B (2010). Physiological complexity and system adaptability: evidence form postural control dynamics of older adults. J. Appl. Physiol..

[20] Captur G (2017). The fractal heart – embracing mathematics in the cardiology clinic. Nat. Rev. Cardiol..

[21] Mäkikallio TH (2001). Prediction of sudden cardiac death by fractal analysis of heart rate variability in elderly subjects. J. Am. Coll. Cardiol..

[22] Bigelow KE, Berme N (2011). Development of a protocol for improving the clinical utility of posturography as a fall-risk screening tool. J. Gerontol. A Biol. Sci. Med. Sci..

[23] Peng CK (2002). Quantifying fractal dynamics of human respiration: age and gender effects. Ann. Biomed. Eng..

[24] Lodha N, Naik SK, Coombes SA, Cauraugh JH (2010). Force control and degree of motor impairments in chronic stroke. Clin. Neurophysiol..

[25] Carver NS, Bojovic D, Kelty-Stephen DG (2017). Multifractal foundations of visually-guided aiming and adaptation to prismatic perturbation. Hum. Mov. Sci..

[26] Jordan K, Challis JH, Newell KM (2007). Walking speed influences on gait cycle variability. Gait Posture.

[27] Slifkin AB, Eder JR (2014). Fitts’ index of difficulty predicts the 1/f structure of movement amplitude time series. Exp. Brain Res..

[28] Conrad, M. *Adaptability: the significance of variability from molecule to ecosystem* Plenum Ress, New York (1983).

[29] Ulanowicz RE (2002). The balance between adaptability and adaptation. BioSystems.

[30] Lang PO, Michel JP, Zekry D (2009). Frailty syndrome: a transitional state in a dynamic process. Gerontology.

[31] Canguilhem, G. *Le normal et le pathologique* Presses Universitaires de France (2013).

[32] Delignières D (2006). Fractal analyses for ‘short’ time series: a re-assessment of classical methods. J. Math. Psychol..

[33] Pierrynowski MR (2005). Reliability of the long-range power-law correlations obtained from the bilateral stride intervals in asymptomatic volunteers whilst treadmill walking. Gait Posture.

[34] Peng CK, Havlin S, Stanley HE, Goldberger AL (1995). Quantification of scaling exponents and crossover phenomena in non stationary heartbeat time series. Chaos.

[35] Almurad Z, Delignières D (2016). Evenly spacing in Detrended Fluctuation Analysis. Physica A.

[36] Liddy JJ, Haddad JM (2018). Evenly spaced Detrended Fluctuation Analysis: selecting the number of points for the diffusion plot. Physica A.

[37] Kantelhardt JW (2002). Multifractal detrended fluctuation analysis of nonstationary time series. Physica A.

[38] Mukli P, Nagy Z, Eke A (2015). Multifractal formalism by enforcing the universal behavior of scaling functions. Physica A.

[39] Wing AM, Kristofferson AB (1973). The timing of interresponse intervals. Percept. Psychophys..

[40] Billon M, Semjen A, Cole J, Gauthier G (1996). The role of sensory information in the production of periodic finger-tapping sequences. Exp. Brain. Res..

[41] Drewing K (2004). Timing of bimanual movements and deafferentation: implications for the role of sensory movement effects. Exp. Brain. Res..

[42] Drewing K (2013). Delayed auditory feedback in repetitive tapping: a role for the sensory goal. Q. J. Exp. Psychol..

[43] Studenka BE, Eliasz KL, Shore DI, Balasubramaniam R (2013). Crossing the arms confuses the clocks: sensory feedback and the bimanual advantage. Psychon. Bull. Rev..

[44] LaRue J (1995). Is proprioception important for the timing of motor activities?. Can. J. Physiol. Pharmacol..

[45] Zelaznik HN (2012). Motor timing deficits in children with attention-deficit/hyperactivity disorder. Hum. Mov. Sci..

[46] Stenneken P (2006). The effect of sensory feedback on the timing of movements: evidence form deafferented patients. Brain Res..

[47] Stenneken P, Prinz W, Bosbach S, Aschersleben G (2006). Visual proprioception in the timing of movements: evidence from deafferentation. NeuroReport.

[48] Vanneste S, Pouthas V, Wearden JH (2001). Temporal control of rhythmic performance: a comparison between young and old adults. Exp. Aging Res..

[49] Merabet LB, Pascual-Leone A (2010). Neural reorganization following sensory loss: the opportunity of change. Nat. Rev. Neurosci..

[50] Weiss T (2004). Rapid functional plasticity in the primary somatomotor cortex and perceptual changes after nerve block. Eur. J. Neurosci..

[51] Ihlen EAF, Vereijken B (2010). Interaction-dominant dynamics in human cognition: beyond 1/*f*^α^ fluctuation. J. Exp. Psychol. Gen..

[52] Kelty-Stephen DG, Wallot S (2016). Multifractality Versus (Mono-) Fractality as Evidence of Nonlinear Interactions Across Timescales: Disentangling the Belief in Nonlinearity From the Diagnosis of Nonlinearity in Empirical Data. Ecol. Psychol..

[53] Ivanov PC, Chen Z, Hu K, Stanley E (2004). Multiscale aspects of cardiac control. Physica A.

[54] Vergotte G (2018). Concurrent changes of brain functional connectivity and motor variability when adapting to task constraints. Front. Physiol..

[55] Merabet LB (2008). Rapid and reversible recruitment of early visual cortex for touch. PLoS one.

[56] Mohan A, Vanneste S (2017). Adaptive and maladaptive neural compensatory consequences of sensory deprivation – From a phantom percept perspective. Prog. Neurobiol..

[57] Kelty-Stephen DG, Palatinus K, Saltzman E, Dixon JA (2013). A tutorial on multifractality, cascades, and interactivity for empirical time series in ecological science. Ecol. Psychol..

[58] Vaillancourt DE, Newell KM (2002). Changing complexity in human behavior and physiology through aging and disease. Neurobiol. Aging.

[59] Conrad M (1977). Functional significance of biological variability. B. Math. Biol..

[60] Cole, J. & Paillard, J. Living without Touch and Peripheral Information about Body Position and Movement: Studies with Deafferented Subjects. *The body and the self*, eds Bermudez J. L., Marcel A., Eilan N. (MIT press, Cambridge), pp 245–266 (1995).

[61] Cole JD (1995). Evoked potentials in a deafferented subject. Can. J. Physiol. Pharm..

[62] Cole JD, Sedgwick EM (1992). The perceptions of force and of movement in a man without large myelinated sensory afferents below the neck. J. Physiol..

[63] Fraisse P (1966). L’Anticipation de stimulus rythmiques. Vitesse d’établissement et précision de la synchronisation. Ann. Psychol..

[64] Finney SA, Warren WH (2002). Delayed auditory feedback and rhythmic tapping: evidence for a critical interval shift. Percept. Psychophys..

[65] Eke A (2000). Physiological time series: distinguishing fractal noises from motions. Pflugers Arch..

[66] Makowiec D (2011). Reading multi-fractal spectra: aging by multifractal analysis of heart rate. EPL.

